# A Novel Anti-Hepatitis C Virus and Antiproliferative Agent Alters Metabolic Networks in HepG2 and Hep3B Cells

**DOI:** 10.3390/metabo7020023

**Published:** 2017-06-02

**Authors:** Adrian Keogh, Sevil Şenkardeş, Jeffrey R. Idle, Ş. Güniz Küçükgüzel, Diren Beyoğlu

**Affiliations:** 1 Visceral and Transplantation Surgery, Department of Clinical Research, University of Bern, 3008 Bern, Switzerland; adrian.keogh@dkf.unibe.ch; 2 Faculty of Pharmacy, Department of Pharmaceutical Chemistry, Marmara University, Haydarpaşa, 34668 Istanbul, Turkey; sevil.aydin@marmara.edu.tr (S.Ş.); gkucukguzel@marmara.edu.tr (Ş.G.K.); 3 Hepatology Research Group, Department of Clinical Research, University of Bern, 3008 Bern, Switzerland; jeffidle@gmail.com

**Keywords:** diflunisal, hydrazone, antihepatitis C virus, antiproliferative, metabolomics, metabolic networks

## Abstract

A series of novel diflunisal hydrazide-hydrazones has been reported together with their anti-hepatitis C virus and antiproliferative activities in a number of human hepatoma cell lines. However, the mechanisms underlying the efficacy of these agents remain unclear. It was chosen to investigate the lead diflunisal hydrazide-hydrazone, 2′,4′-difluoro-4-hydroxy-*N*′- [(pyridin-2-yl)methylidene]biphenyl-3-carbohydrazide (compound 3b), in two cultured human hepatoma cell lines—HepG2 and Hep3B—using a metabolomic protocol aimed at uncovering any effects of this agent on cellular metabolism. One sub-therapeutic concentration (2.5 μM) and one close to the IC_50_ for antimitotic effect (10 μM), after 72 h in cell culture, were chosen for both compound 3b and its inactive parent compound diflusinal as a control. A GCMS-based metabolomic investigation was performed on cell lysates after culture for 24 h. The intracellular levels of a total of 42 metabolites were found to be statistically significantly altered in either HepG2 or Hep3B cells, only eight of which were affected in both cell lines. It was concluded that compound 3b affected the following pathways—purine and pyrimidine catabolism, the glutathione cycle, and energy metabolism through glycolysis and the pentose phosphate pathway. Although the metabolomic findings occurred after 24 h in culture, significant cytotoxicity of compound 3b to both HepG2 and Hep3B cells at 10 μM were reported not to occur until 72 h in culture. These observations show that metabolomics can provide mechanistic insights into the efficacy of novel drug candidates prior to the appearance of their pharmacological effect.

## 1. Introduction

It is now well understood that the vast majority of diseases involve altered patterns of cellular metabolites. Cancer has been well studied in this regard, particularly with respect to energy metabolism. Glucose, glutamine, acetate, lactate, pyruvate, hydroxybutyrate, and fatty acids may all be metabolized faster in malignant tissues than in their non-tumor counterparts [1]. Altered metabolism in cancer cells is viewed as now providing potentially new therapeutic targets [2]. Moreover, these so-called metabolic hallmarks of cancer cells are believed to lead to an altered interplay between immune cells and tumor cells that can lead to immunosuppression. The view has been expressed that metabolic reeducation of cancer cells may not only assist in direct tumor cytotoxicity, but also attenuate metabolic immunosuppression in the tumor microenvironment, thus facilitating immunotherapy [3]. In addition, lipid signaling pathways can also be altered in various cancers. For example, we have recently reported that a significant number of lipid groups, including prostaglandins and their precursors, phosphocholines, fatty acids, sphingolipids, triglycerides, together with cholesterol and its esters, are all altered in the plasma of patients presenting with acute myeloid leukemia [4]. Finally, chronic viral infection that can lead to cancer, such as with hepatitis C virus (HCV), can provoke profound metabolic alterations in hepatocytes [5] that can be detected in plasma and urine through metabolomic investigation [6]. Understanding how host cell metabolic pathways can be hijacked by oncogenic viruses and ultimately by cancer cells should offer new avenues for therapy. Furthermore, insights into the effects of new therapeutic agents on the metabolism of virus-infected cells and tumors is expected to lead to improved therapeutic solutions.

HCV infection is a major global health problem with an estimated 150–160 million people assumed to be infected, approximately 3% of the world’s population [7,8]. Chronic hepatitis C leads to fibrotic changes in the liver, and ultimately to cirrhosis, after the development of which, the patient has a high risk of developing hepatocellular carcinoma (HCC). The goal of HCV treatment has been to eradicate the infection, thereby terminating the chronic liver inflammation and, thus, decreasing the complications that can follow cirrhosis [7]. Interferon-α (IFN) combined with ribavirin (RBV) was the mainstay of HCV treatment but more recently so-called direct acting antivirals (DAAs) have been developed that have, in part, replaced IFN/RBV-based treatments. The first generation comprised protease inhibitors (boceprivir and telaprevir), then the HCV NS5B RNA polymerase inhibitor (sofosbuvir) and the NS5A replication complex inhibitor (daclatasvir) [8]. Resistance to DDAs is a growing clinical problem that is incompletely understood [9]. Accordingly, there is clearly a need for the development of further clinically- and cost-effective anti-HCV drugs with a high threshold to resistance. Additionally, chemotherapeutic options for HCC are limited. Many clinical trials have ended in failure [10]. The most commonly used agent is sorafenib, which is a drug that inhibits serine-threonine kinases Raf-1 and B-Raf, the receptor tyrosine kinase activity of vascular endothelial growth factor receptors (VEGFRs) 1, 2, and 3, and platelet-derived growth factor receptor b (PDGFR-b) [11,12]. Sorafenib, therefore, inhibits both tumor cell proliferation and angiogenesis. New and effective candidate chemotherapeutic agents for the treatment of HCC are also required.

**Figure 1:**
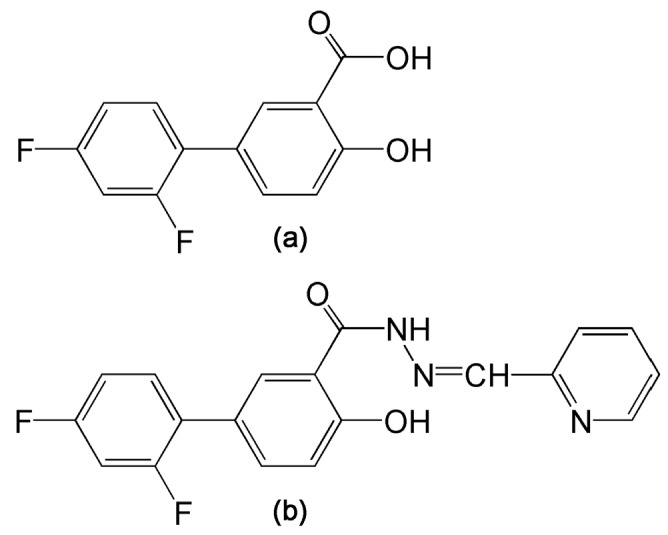
(**a**) Diflunisal; (**b**) 2′,4′-Difluoro-4-hydroxy-*N*′-[(pyridin-2-yl)methylidene]biphenyl-3-carbohydrazide (compound 3b).

A series of novel derivatives of the non-steroidal anti-inflammatory drug (NSAID) and the analgesic agent diflunisal have been reported that possess both inhibition of HCV replication and dose-dependent antiproliferative activity in a range of hepatoma cell lines [13]. Diflunisal derivatives have frequently been reported to possess several biological activities, including anticancer, anti-HIV, anticonvulsant, antimicrobial, and anti-inflammatory properties [13]. Of this series, 2′,4′-difluoro-4-hydroxy-*N*′-[(pyridin-2-yl)methylidene]biphenyl-3-carbohydrazide (termed compound 3b; Figure 1) displayed IC_50_ values in the 5–15 μM range for cell cycle arrest and apoptotic cell death in six hepatoma cell lines [13]. Currently, the mechanisms that underlie these effects are unknown. We have, therefore, conducted a gas chromatography-mass spectrometry (GCMS)-based metabolomic investigation of compound 3b and diflunisal in two HCC-derived cell lines, HepG2 and Hep3B in order to discover metabolic pathways that might be perturbed in relation to the antiproliferative and proapoptotic properties of compound 3b. We chose not to study drug effects on primary human hepatocytes (PHHs) since there were no data in the original study [13]. In addition, PHHs from different donors have been reported to display wide interindividual variation in metabolic activity [14] that also can vary considerably with respect to culture time, even in the first 24 h [15]. Metabolomic data collected in HepG2 and Hep3B hepatoma cell lines are expected to reveal novel insights into the pharmacological effects of the lead compound 3b, which is intended to be active against hepatocellular carcinoma. When healthy liver tissue is the disease target, as with paracetamol/acetaminophen toxicity, studies in PHHs are more applicable [14].

## 2. Results

### 2.1. Metabolomic Analysis—Compound 3b versus Diflunisal

**Figure 2:**
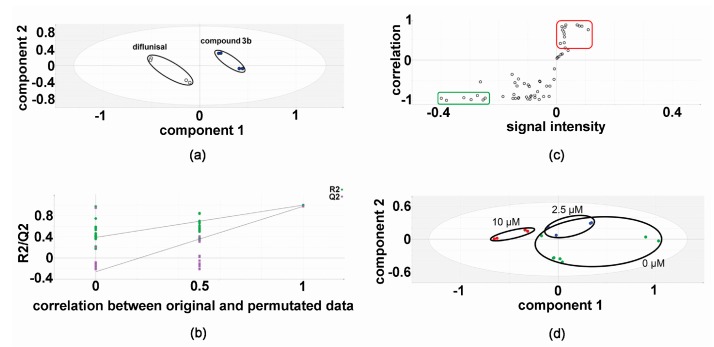
Metabolomic analysis of HepG2 cellular metabolites after treatment with compound 3b and diflunisal in discovery experiments. (**a**) PLS-DA score plot showing clustering and separation of metabolomic data from four culture flasks with compound 3b and four with diflunisal (control); (**b**) leave-one-out cross-validation of PLS-DA model where R2 is the correlation coefficient and Q2 the predictability coefficient; (**c**) OPLS-DA loadings S-plot where each point represents a metabolite and the red and green boundary boxes enclose cellular metabolites that are up- and downregulated, respectively, by compound 3b; and (**d**) PLS-DA scores plot for HepG2 cellular metabolites after treatment with compound 3b at 0, 2.5, and 10 μM.

**Figure 3:**
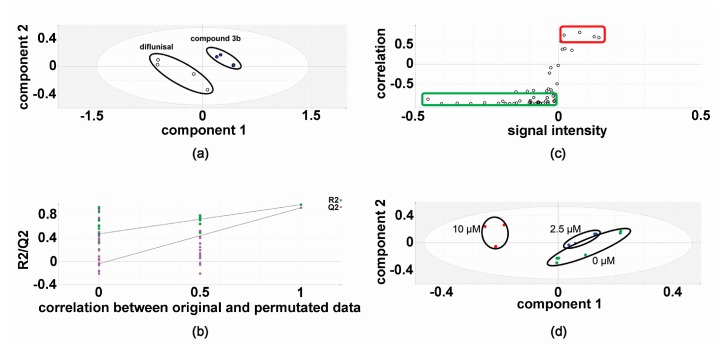
Metabolomic analysis of Hep3B cellular metabolites after treatment with compound 3b and diflunisal. (**a**) PLS-DA score plot showing clustering and separation of metabolomic data from four culture flasks with compound 3b and four with diflunisal (control); (**b**) leave-one-out cross-validation of PLS-DA model where R2 is the correlation coefficient and Q2 the predictability coefficient; (**c**) OPLS-DA loadings S-plot where each point represents a metabolite and the red and green boundary boxes enclose cellular metabolites that are up- and downregulated, respectively, by compound 3b; and (**d**) PLS-DA scores plot for HepG2 cellular metabolites after treatment with compound 3b at 0, 2.5, and 10 μM.

In the discovery experiments, metabolomic analysis of compound 3b versus diflunisal (four flasks vs. four flasks) at 10 μM in both HepG2 and Hep3B cells revealed clustering and separation of the two treatments in the projection to latent structures-discriminant analysis (PLS-DA) scores plot for HepG2 cells (Figure 2) [6,16,17]. A leave-one-out cross-validation [6,16,17] was conducted to determine that the PLS-DA model was valid (Figure 2). Orthogonal PLS-DA (OPLS-DA) analysis was performed to reveal the differential metabolites that drove the separation of compound 3b and diflunisal metabolomic phenotypes in Figure 2. An OPLS-DA loadings S-plot (Figure 2) [6,16,17] shows cellular metabolites that were both upregulated and attenuated in HepG2 cells by treatment with compound 3b, relative to diflunisal treatment. These metabolite levels were subjected to univariate statistics and the results are shown in Table 1, combined with validation findings for 10 μM diflunisal (control) vs. 10 μM compound 3b (5 vs. 5). Similarly, the metabolite data from treated Hep3B cells was also subjected to PLS-DA (Figure 3) and OPLS-DA (Figure 3) analysis. It is noteworthy that the PLS-DA score plots for HepG2 (Figure 2) and Hep3B (Figure 3) are similar. However, in the OPLS-DA loadings S-plots (Figure 2 and Figure 3), there occurred more upregulated metabolites for the HepG2 than the Hep3B cell incubations with compound 3b and, conversely, more downregulated metabolites for Hep3B than HepG2 incubations. The augmented and diminished cellular metabolites in Hep3B cells were also subjected to univariate statistical analysis with the results given in Table 1 alongside the HepG2 findings.

**Figure 4:**
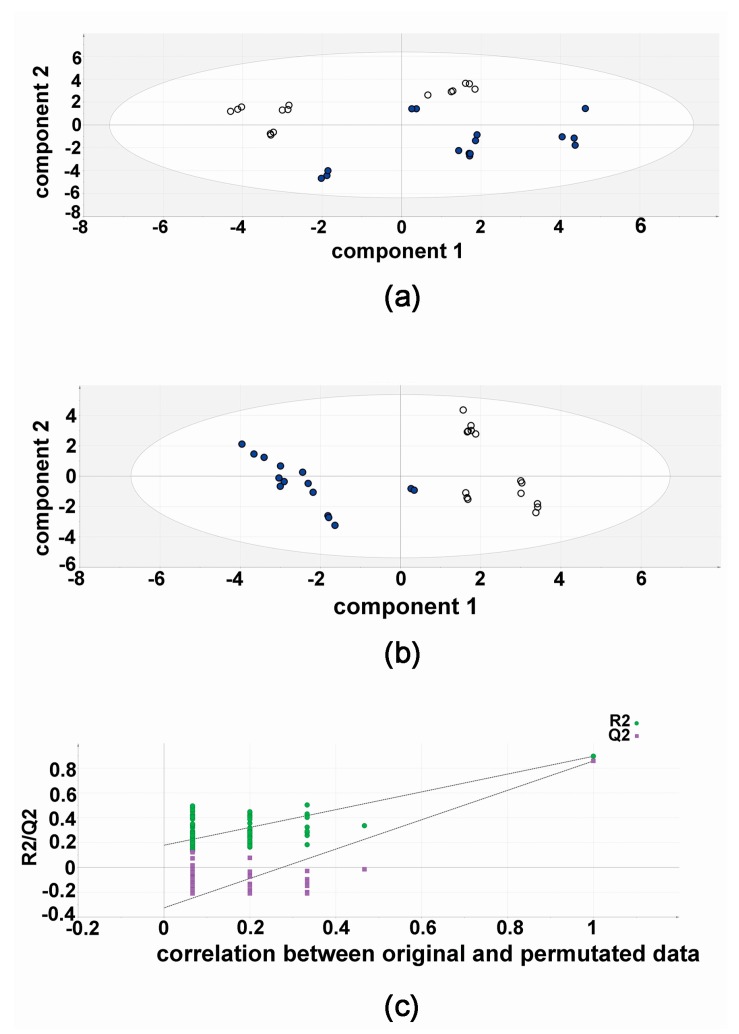
Metabolomic analysis of HepG2 cellular metabolites after treatment with compound 3b and diflunisal in validation experiments. (**a**) Principal components analysis (PCA) scores plot showing the separation of metabolomic data from five culture flasks with compound 3b and five with diflunisal (control), each analyzed in triplicate. Filled symbols represent compound 3b and open symbols represent diflunisal; (**b**) PLS-DA scores plot for HepG2 cellular metabolites after treatment with compound 3b at 10 μM in validation experiments. Filled symbols represent compound 3b and open symbols represent diflunisal; (**c**) leave-one-out cross-validation of PLS-DA model where R2 is the correlation coefficient and Q2 is the predictability coefficient, showing that the PLS-DA model is valid.

The results of the validation experiments, five flasks of HepG2 with both compound 3b and diflunisal, each analyzed in triplicate (15 vs. 15) are shown in Figure 4. The PCA scores plot shows a separation of compound 3b and diflunisal metabolic effects on HepG2 cells. In this unsupervised analysis, the differential metabolic effects of compound 3b and diflunisal on HepG2 cells at 10 μM can clearly be seen. As in the discovery experiment, compound 3b and diflunisal are separated in the PLS-DA score plot (Figure 4). The cross-validation analysis (Figure 4) shows that the PLS-DA model was valid.

Forty-two cellular metabolites were statistically significantly altered in HepG2 and Hep3B cells, of which 38 eluted in the GCMS as single peaks and three (ethanolamine, glycine, and lysine) as two peaks due to double chemical derivatives and one (galactose) as two peaks due to formation of *O*-methyloxime (*R*)- and (*S*)-geometric isomers. In these cases, peak areas were summed to yield PAR values for multivariate data analysis. In HepG2 cells, seven cellular metabolites were statistically significantly attenuated and 16 were statistically significantly upregulated (Table 1). The pattern of affected cellular metabolites was somewhat different in Hep3B, with 23 cellular metabolites attenuated, but only four (glyceric acid, fructose 6-phosphate, glucose 6-phosphate, and guanosine) upregulated. Interestingly, two of these metabolic upregulations (glucose 6-phosphate and fructose 6-phosphate) were also observed in HepG2 cells. Of the 23 metabolites attenuated by compound 3b in Hep3B cells, only one (creatinine) was also attenuated by compound 3b in HepG2 cells. 

### 2.2. Metabolic Networks Modified by Compound 3b

Untargeted metabolomic analyses were employed to infer which metabolic networks were modified by compound 3b in cell culture. Additionally, since a larger number of upregulated metabolites were found in HepG2 cells compared to Hep3B cells, most insights into metabolic networks derived from the HepG2 data.

#### 2.2.1. Purine and Pyrimidine Metabolism

**Figure 5:**
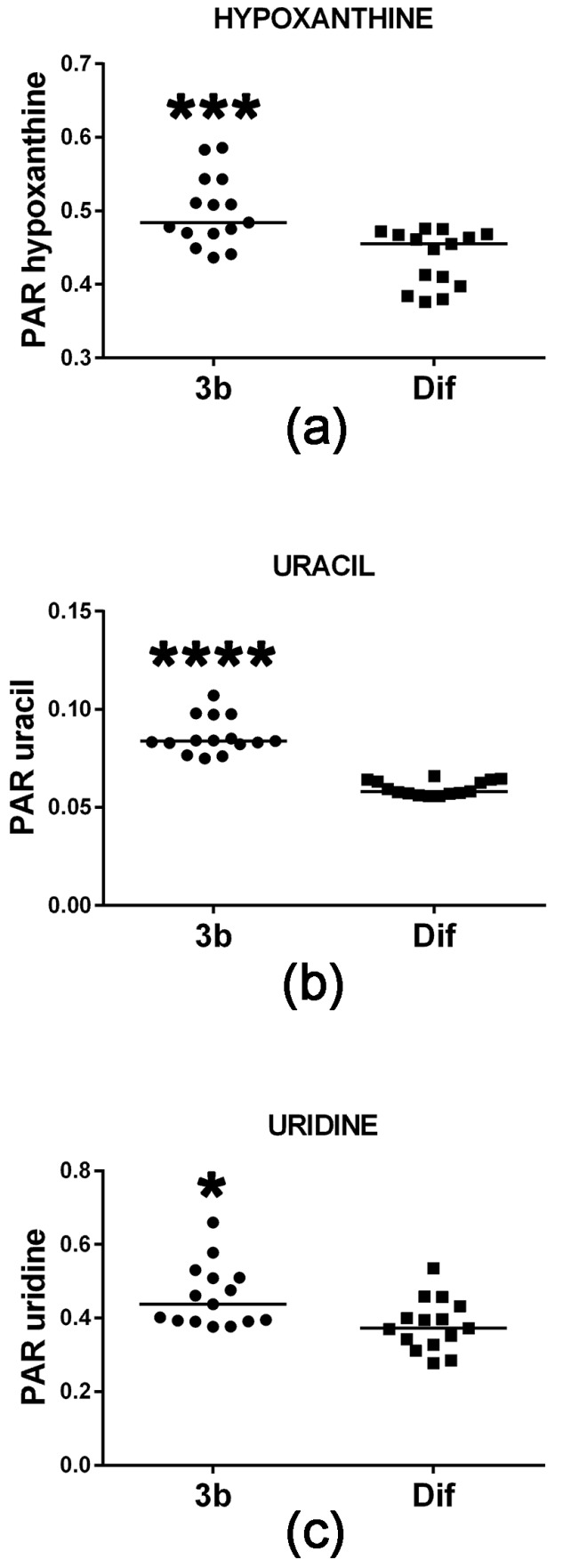
Upregulated purine and pyrimidine metabolism in HepG2 cells by compound 3b. PAR means peak area ratio (relative concentration). Horizontal lines represent the medians. * means *p* < 0.05; *** means *p* < 0.001; and **** means *p* < 0.0001.

Cell proliferation requires de novo synthesis of purine and pyrimidine nucleotides to sustain not only DNA replication, but also the RNA synthesis, which is required for protein synthesis, particularly during the S-phase of the cell cycle [19]. This involves a high turnover of ATP and proliferating cells usually maintain ATP concentrations above 1 mM [19,20] to provide both cellular energy and for the synthesis of precursors of RNA and DNA. When cells do not maintain sufficient ATP synthesis by mitochondrial oxidative phosphorylation, they can fail to complete the cell cycle by stalling at either G1 or at G2-M [21]. Conversely, it is expected that agents which reduce cell proliferation will concomitantly diminish the cellular ATP pool. Figure 5 reveals that compound 3b treatment of HepG2 cells increased intracellular levels of the ATP metabolite hypoxanthine. Although statistically significant, this increase was +6% and, thus, may be of minor biological significance. Although intracellular concentrations of the two purines xanthine and guanine were also determined in this study, they were unaltered by compound 3b treatment. The pyrimidine uracil and its ribonucleoside uridine were also increased in HepG2 cells by compound 3b treatment (Figure 5), by +31% and +15%, respectively. It is unlikely that this is due to de novo pyrimidine synthesis since this is closely tied to the activity of the cell cycle [22]. It is more likely to result from increased RNA turnover and nucleoside salvage as cells enter apoptosis in response to compound 3b [13].

#### 2.2.2. Glutathione Synthesis and Breakdown

**Figure 6:**
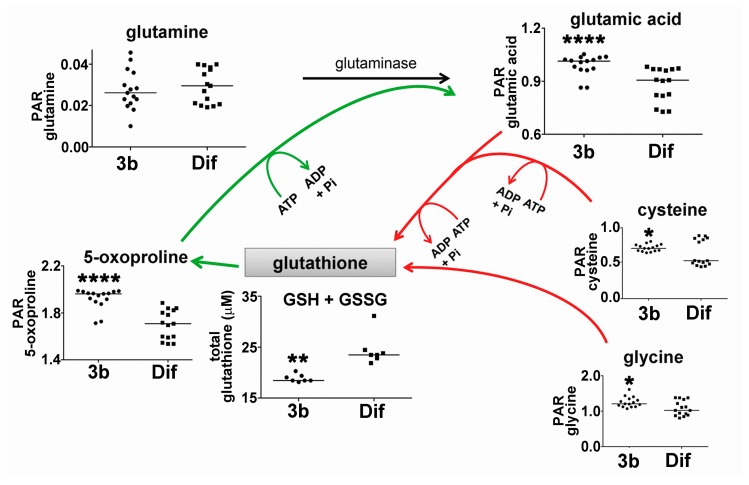
Upregulation of the glutathione cycle in HepG2 cells by compound 3b. Anabolic reactions are shown in red. Catabolic reactions are shown in green. PAR means peak area ratio (relative concentration). Horizontal lines represent medians. * means *p* < 0.05; ** means *p* < 0.01; and **** means *p* < 0.0001.

Reduced glutathione (GSH, l-γ-glutamyl-l-cysteinylglycine) is a tripeptide that is formed from glutamic acid, cysteine, and glycine that acts as an antioxidant and reducing agent controlling the cellular redox state. It has also been proposed that glycine may protect the cell by conjugating potentially toxic acids and making them available for deportation [23,24]. The presence of reactive oxygen species (ROS) depletes GSH, oxidizing it to its dimer GSSG, which can be reduced back to GSH by the action of glutathione reductase (GR). Over-expression of GR in HepG2 cells has been reported to ameliorate levels of ROS generated from H_2_O_2_ and restore GSH levels and cellular viability by impairing apoptosis [25]. Studies have established that GSH depletion and post-translational modification of proteins by glutathionylation are critical regulators of apoptosis [26]. Figure 6 shows the sequential addition of glutamic acid, cysteine and glycine in the de novo synthesis of GSH. Intracellular concentrations of all three amino acid precursors in HepG2 cells were statistically significantly enhanced by compound 3b, by +10.7%, +21.9%, and +15.3%, respectively. Furthermore, 5-oxo-proline, which is a metabolite of GSH that is recycled to glutamic acid [27], was also enhanced +13% in HepG2 cells. As Figure 6 shows, glutathione anabolism and catabolism both appear to have been upregulated by compound 3b. It should be noted that three of the reactions shown in Figure 6 require ATP and, therefore, may contribute to increased ATP and adenine turnover, with concomitant elevation of cellular hypoxanthine (Figure 5).

The effect of compound 3b on cellular levels of total glutathione (reduced, GSH and oxidized, GSSG) in HepG2 cells was also investigated. Interestingly, total glutathione in compound 3b-treated cells was statistically significantly lower than in diflunisal-treated cells (Figure 5). Presumably, compound 3b increased glutathione catabolism (green arrows) at the expense of glutathione anabolism (red arrows) leading to increased intracellular 5-oxoproline and glutamic acid. It should be noted that the mean difference in 5-oxoproline concentration was greater than the differences in the GSH precursors glutamate, cysteine, and glycine (Figure 6).

#### 2.2.3. Energy Metabolism by Glycolysis and the Pentose Phosphate Pathway

**Figure 7:**
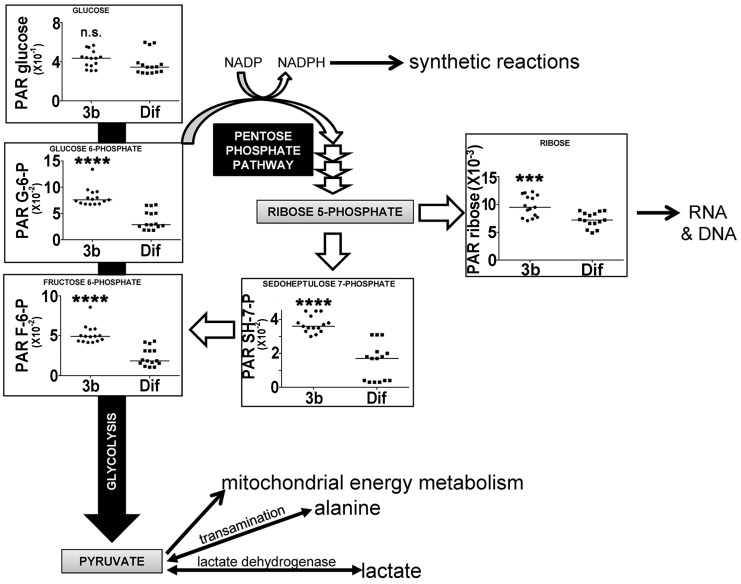
Upregulation of glycolysis and the pentose phosphate pathway by compound 3b in HepG2 cells leading to increased ribose synthesis. PAR means peak area ratio (relative concentration). Horizontal lines represent medians. n.s. means not statistically significantly different; *** means *p* < 0.001; and **** means *p* < 0.0001.

Glycolysis is a non-oxygen-requiring, energy-generating pathway converting glucose to pyruvate through a number of intermediates, the first two of which are glucose 6-phosphate (G6P) and fructose 6-phosphate (F6P) [28]. When pyruvate is subsequently used for mitochondrial oxidation, the process is referred to as mitochondrial oxidation. When pyruvate is reduced instead to lactate or transaminated to alanine, this pathway is known as aerobic glycolysis, as happens in malignant tumors and cancer cell lines, often referred to as the Warburg effect. HepG2 cells have the capacity for aerobic glycolysis [17]. Glycolysis also serves as the starting point for the pentose phosphate pathway (PPP), which generates NADPH for use in biosynthetic reactions and also pentoses, such as ribose [29]. Figure 7 shows that glycolytic intermediates G6P and F6P are upregulated in HepG2 cells by compound 3b, both by +62%. These glycolytic intermediates were also upregulated by compound 3b in Hep3B cells (Table 1). In the PPP, ribose was upregulated +24% by compound 3b in HepG2 cells, while sedoheptulose 7-phosphate was increased +53% in HepG2 cells and +12.7% in Hep3B cells. There were no statistically significant differences in the cellular concentrations of the glycolytic end-products pyruvate, lactate, or alanine due to compound 3b treatment of either cell line. Moreover, none of the measured tricarboxylic acid cycle intermediates [29], citrate, succinate, or 2-oxoglutarate, were altered by compound 3b treatment, suggesting no effect on mitochondrial oxidation of pyruvate. The effects observed, therefore, appeared to be restricted to the proximal end of glycolysis and the PPP. However, it is conceded that the elevation in cellular ribose concentration, like uracil and uridine, may be due to the salvage of RNA components after nucleic acid digestion in apoptosis or autophagy.

#### 2.2.4. Pantothenic Acid Transport and Fatty Acid β-Oxidation

**Figure 8:**
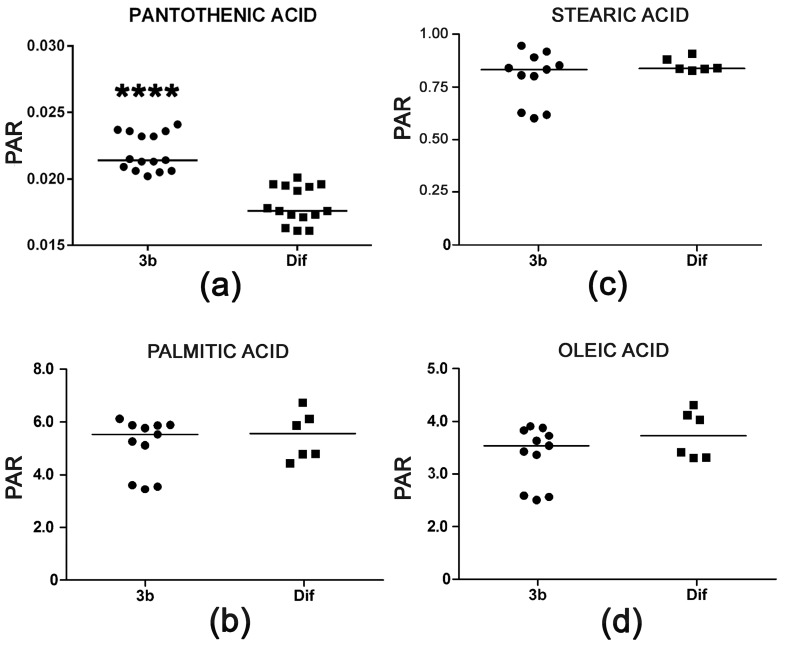
(**a**) Increased cellular concentration of pantothenic acid with no concomitant reduction in fatty acid concentrations in HepG2 cells after treatment with compound 3b relative to diflunisal. Intracellular concentrations of palmitic acid (**b**), stearic acid (**c**), and oleic acid (**d**) were unaltered. Horizontal lines represent medians. **** means *p* < 0.0001.

Pantothenic acid is a mammalian vitamin that is transported into cells by a multivitamin transporter. It is present in the cell culture medium employed. The role of pantothenic acid is as an obligatory precursor for coenzyme A, which is used in many metabolic reactions, including the degradation of fatty acids by mitochondrial β-oxidation. The observed increase of +18% pantothenic acid in HepG2 cells treated with compound 3b appeared not to be associated with increased β-oxidation of fatty acids that might be due to increased coenzyme A synthesis. Palmitic, stearic, and oleic acid cellular concentrations were unaltered (Figure 8). Interestingly, arachidonic acid was increased >40-fold after treatment with diflunisal (data not shown). Diflunisal is a prostaglandin E synthesis inhibitor [30] and, thus, expected to elevate arachidonic acid concentrations. Compound 3b does not share this property of the parent compound.

### 2.3. Effect of Compound 3b and Diflunisal on HepG2 and Hep3B Cell Viability

**Figure 9:**
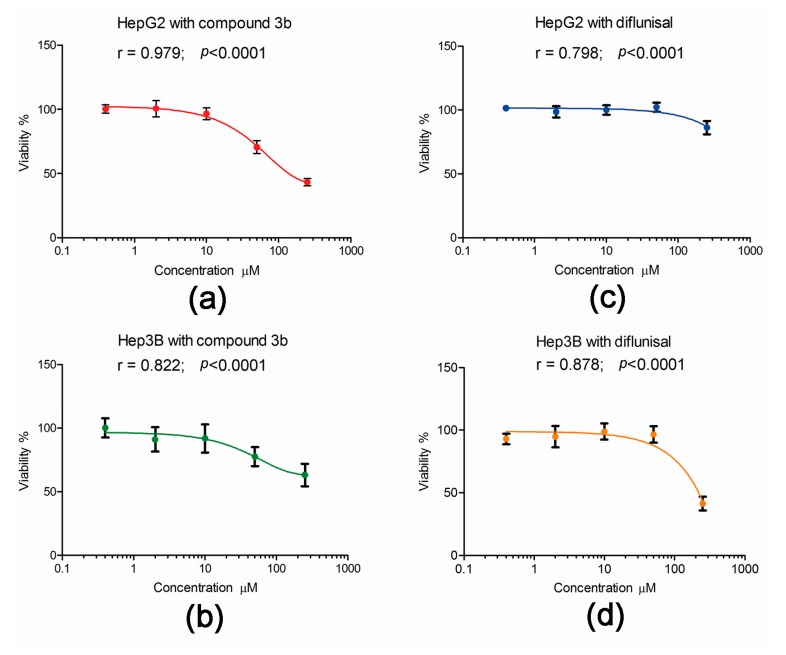
The effect of compound 3b and diflunisal on the viability of HepG2 and Hep3B cell 24 h cultures. In each case, data highly statistically significantly fitted a monophasic exponential decline in viability with increasing concentration. (**a**) Increasing cytotoxicity of compound 3b (0.4–250 μM) with HepG2 cells; (**b**) Increasing cytotoxicity of compound 3b (0.4–250 μM) with Hep3B cells; (**c**) a plateau cytotoxic response for diflunisal with HepG2 cells; and (**d**) a plateau cytotoxic response for diflunisal with Hep3B cells with cytotoxicity only observed at 250 μM.

The published data on the cytotoxicity of compound 3b and its parent diflunisal were derived from exposure of hepatoma cell lines to the compounds in culture for 72 h [13]. To examine the effect of these two compounds on the viability of HepG2 and Hep3B cells under the conditions used in the metabolomic investigations, cell viability after 24 h exposure was conducted. As shown in Figure 9, the effect of both compound 3b and diflunisal was less after 24 h than the published values of cytotoxicity at 72 h [13]. This is not surprising since the doubling times in our hands of HepG2 and Hep3B cells were 40 h and 25 h, respectively. Compound 3b was cytotoxic to both HepG2 and Hep3B cells (Figure 9), showing a monophasic exponential decline in cell viability over the range of concentrations, 0.4, 2.0, 10.0, 50.0, and 250 μM. However, at the concentration used in the metabolomic experiments (10 μM), cell viability at 24 h was >90%. In contrast, diflunisal only showed toxicity at 250 μM, with a plateau in cell viability from 0.4 to 50.0 μM. Although the differences in cytotoxicity after 24 h between compound 3b and diflunisal were difficult to observe at 10 μM, clear perturbations of the cellular metabolome in both cell lines were observed at this concentration. This underscores the power of metabolomics in revealing changes in cell phenotype that might not be apparent at the whole cell level.

## 3. Discussion

In an attempt to address perceived issues in the pharmaceutical industry, it has recently been proposed to include metabolomics as a “routine component of the drug discovery process…to provide feedback on the in vivo mechanism of action” [31]. A workflow using NMR-based metabolomics was proposed that was envisaged as improving the efficiency and success rate of drug discovery [31]. These concepts have been further developed in which metabolomics was envisaged to have utility in biomarker identification, mechanism of action, drug candidate efficacy and safety, patient selection and stratification, and treatment and patient follow-up, all assisting in the drug discovery and development process [32]. It is in the spirit of these recent discussions that we undertook a metabolomic investigation of the lead molecule compound 3b compared to its parent molecule diflunisal. Additionally, we used target cells for this molecule, two hepatoma cell lines with widely different cell biologies [33]—HepG2 and Hep3B—in an effort to unmask metabolic information pertinent to this drug’s mode of action.

The lead molecule compound 3b affected metabolic pathways differently in the two hepatoma cell lines. There are many examples of differential responses to drugs by these two cell types. The underlying mechanisms of this have been discussed in detail [33] and are in part based upon the observations that HepG2 is hepatitis B virus (HBV)-negative and non-tumorigenic, while Hep3B is HBV-positive and tumorigenic. Moreover, RAS signaling is low in HepG2 and high in Hep3B. Additionally, HepG2 cells are positive for the expression of the p53 tumor suppressor protein but negative for expression of cyclooxygenase 2 (COX2). In contrast, Hep3B cells do not express p53 protein but do express COX2. Many of the differences in gene expression in these two cell lines are due to the HBx protein, a pleiotropic regulator of gene expression involved in the development of HCC [33].

The issue of dose-dependency of the metabolic effects of compound 3b was addressed. Both HepG2 and Hep3B cells responded to compound 3b in a dose-dependent manner. The metabolomic phenotype for 2.5 μM compound 3b overlapped with the metabolomic phenotype of untreated cells in both cell lines, while the metabolomic phenotype for 10 μM compound 3b was distinct from the control and 2.5 μM phenotypes for both cell lines. These findings, which represent the metabolic reprogramming of hepatoma cells when cultured with compound 3b, are in agreement with the published dose-dependency efficacy data for the antiproliferative effects of compound 3b in both cell lines [13]. Here, the metabolomic data garnered for compound 3b was in agreement with the limited information regarding its biological properties.

These investigations produced novel insights into the pharmacology of compound 3b beyond its dose-dependent effects in HepG2 and Hep3B cells. It is surprising that so few of the metabolic perturbations elicited by the drug were common to both cell types. Moreover, in most cases when a metabolite was altered in both cell types, the direction of change was converse (5-oxoproline, cysteine, glutamate, lysine, and tyrosine). In only three cases was a metabolite altered in the same direction in both cell lines (creatinine, F6P, and G6P). This may indicate that compound 3b has an effect on both the urea cycle (where creatinine is ultimately synthesized from arginine) and glycolysis and the pentose phosphate pathway (where G6P and F6P are produced). This underscores the importance of using more than one cell culture model in metabolomic investigations.

As has been stated earlier, pantothenic acid is not produced by mammalian cells, but rather is a vitamin that is transported into HepG2 cells by a sodium-dependent multivitamin transporter (SMVT) [34,35,36] that is encoded by the *SLC5A6* gene [36]. It is surmised that compound 3b increases CoA synthesis by enhancing SLC5A6 activity to increase pantothenic acid transport from the culture medium, where it is present at a concentration of 16.8 μM. SLC5A6 accounts for 98.6% of pantothenic acid uptake across the human blood-brain barrier and has been studied in detail in a human microvascular endothelial cell line [37]. The most potent drug inhibitors of SLC5A6 were reported to be NSAIDs, including indomethacin, ketoprofen, diclofenac, ibuprofen, phenylbutazone, and flurbiprofen [37]. Diflunisal is an NSAID and, thus, is therefore potentially a greater inhibitor of pantothenic acid uptake by HepG2 cells than compound 3b. Given the free fatty acid profiles determined in this investigation, it appears unlikely that the elevated pantothenic acid translated through elevated coenzyme A synthesis to enhanced fatty acid β-oxidation.

An untargeted GCMS-based metabolomic investigation of the effects of compound 3b and diflunisal permitted conclusion that the lead compound upregulated the following pathways—purine and pyrimidine catabolism, the glutathione cycle, together with energy metabolism through glycolysis and the pentose phosphate pathway. The lead compound 3b displayed clear differences in metabolic effects from the parent diflunisal in both HepG2 and Hep3B hepatoma cells. Although both compounds appeared to be similarly cytotoxic with 24 h culture at 10 μM, metabolomics revealed changes in underlying metabolic mechanisms that presaged impending cytotoxicity due to compound 3b. These findings are novel, not only in relation to compound 3b, but also to the effects of drugs on hepatoma cells in general. Future studies of drug effects on cultured tumor cells should include a metabolomic component in order to understand the effect of the drugs on intermediary metabolism, especially if such studies are part of a high-throughput screening protocol within the drug discovery and development process [31,32].

It is very likely that novel antiviral and anticancer drugs leave a unique metabolomic footprint on target cells. This was recently demonstrated in a metabolomic study of the antiproliferative effects of physapubenolide, a withanolide first isolated from *Physalis pubescens* of the nightshade family. Treatment of HepG2 cells with physapubenolide, followed by GCMS-based metabolomic analysis, resulted in the detection of a decreased lactate production, which led the authors to a precise mechanism of action of this potential new drug involving the Akt-p53 pathway [14]. Furthermore, metabolomics has been successfully employed in the discovery of a novel antibiotic, 7-prenylisatin, which was isolated from *Streptomyces* sp. MBT28, following a strategy based upon NMR-based metabolomics. This new drug candidate, which showed activity against *Bacillus subtilis*, was shown to be produced from tryptophan by indole prenyltransferase [15]. Metabolomics can, therefore, provide a new toolbox in the field of medicinal chemistry.

## 4. Materials and Methods

### 4.1. Drugs

Diflunisal and 2′,4′-difluoro-4-hydroxy-*N*′-[(pyridin-2-yl)methylidene]biphenyl-3-carbohydrazide (compound 3b) were previously described [13]. Stock solutions in dimethylsulfoxide (DMSO) were prepared for addition to the cell cultures to yield final concentrations of 0, 2.5 and 10 μM. These concentrations were chosen on the basis of the published IC_50_ values for cell growth inhibition of compound 3b in HepG2 cells and Hep3B cells of 10.3 and 16.2 μM, respectively [13].

### 4.2. Cell Culture, Drug Treatment, and Harvesting of Intracellular Contents

HepG2 and Hep3B cell lines were purchased frozen from ATCC (LCG Standards GmbH, Wesel, Germany). Both cell lines were tested for mycoplasma contamination and only lines testing negative were used in the study. Cells were cultured to 90% confluence in T75 culture flasks in DMEM containing 10% FBS and 100 U/mL penicillin/100 mg/mL streptomycin (Life Technologies, Carlsbad, CA, USA), as described [17,38], then split 3:1 and subcultured in T75 flasks. The concentration of the energy molecules d-glucose and l-glutamine in the medium was 25 and 4 mM, respectively.

In discovery experiments, both HepG2 and Hep3B cells were exposed in culture to compound 3b and diflunisal at 0, 2.5 and 10 μM concentrations for 24 h, with four flasks at each concentration. In validation experiments, a further six flasks of HepG2 cells were exposed in culture for 24 h to 10 μM compound 3b and six flasks of HepG2 cells to 10 μM diflunisal, five of which were used for metabolomic investigations. The cells were then harvested for analysis. Adherent cells were removed by gentle scraping, washed twice with ice-cold PBS, spun, resuspended in ice-cold distilled water and briefly sonicated to liberate water-soluble metabolites as described.

### 4.3. Cell Viability Assay and Cellular Protein Determination

HepG2 and Hep3B cell viability was assessed in culture using a resazurin assay where the virtually nonfluorescent blue resazurin is reduced to the highly-fluorescent compound resorufin by dehydrogenase enzymes in metabolically-active cells, generating a quantitative measure of viability. Briefly, resazurin (0.01 mg/mL) (Sigma-Aldrich) was added to the culture medium and incubated for 1 h. Fluorescence as an indicator of cell viability was measured (excitation 545 nm, emission 590 nm) with a Tecan Infinite^®^ 200 plate reader and I-control software. Untreated cells were used to generate the 100% viability values.

Protein determinations were made by the Bradford method using commercial reagents (Bio-Rad, Cressier, Switzerland) according to the manufacturer’s instructions that yielded a linear calibration (R^2^ = 0.994). Aqueous extracts of HepG2 and Hep3B cells had protein concentrations in the range 2–18 μg/μL.

### 4.4. Gas Chromatography-Mass Spectrometry (GCMS) Analysis

Aliquots of aqueous intracellular extracts (50 μL) were subjected to GCMS analysis with 4-chlorophenylacetic acid (2 mM; 100 μL) as internal standard using a slight modification of our published methods [6,16,17]. Samples were treated with an equal volume of ultrapure pyridine and then blown to dryness under N_2_ and derivatized with methoxyamine hydrochloride (MOX) and BSTFA/TMCS as described [6]. Samples (1.0 μL) were injected in duplicate for the discovery experiment, and in triplicate for the validation experiment using an Agilent 7683B liquid sampler into an Agilent 6890N gas chromatograph with an Agilent 5975B mass selective detector operating under electron impact ionization at 70 eV. The front inlet was operated in splitless mode at 250 °C and an HP5-MS column (60 m; i.d. 250 μm; film thickness 0.25 μm) subjected to a temperature program of 70 °C for 3 min, 10 deg/min to 250 °C, 10 deg/min to 300 °C, held for 8 min (run time 34 min). Mass spectra were collected from m/z 35.0 to 650.0.

In each 34 min chromatogram, in addition to background peaks and the internal standard, 63 peaks deriving from 58 discrete metabolites were identified by comparison of their mass spectra with the NIST 14 Library (MS Wil GmbH, Wil, Switzerland) that contains 276,248 mass spectra from 242,466 compounds and by comparison of their retention times with an in-house collection of 120 authentic standards.

Each annotated peak was quantitated as a peak area ratio (PAR) from its peak area/internal standard peak area using AutoQuant in the on-board Agilent ChemStation software, followed by QuantBrowser GCMS software (Leoson BV, Middelburg, The Netherlands). The resulting spreadsheet of PARs, normalized against protein content, for each annotated metabolite for both cell line lysates treated with diflunisal and compound 3b at each of three concentrations (0, 2.5, and 10 μM) was imported into SIMCA 14 (MKS Data Analytics Solutions, Malmö, Sweden). Multivariate data analysis using unsupervised PCA, supervised PLS-DA, with leave-one-out cross validation of PLS-DA model, and OPLS-DA were conducted as described [6]. Univariate statistics was performed using GraphPad Prism 6.07 (GraphPad Software, Inc., La Jolla, CA, USA). For statistical evaluation of three concentrations (0, 2.5, and 10 μM), analysis of variance with Bonferroni’s multiple comparisons test was used, except where unequal variances occurred, then the nonparametric Kruskal-Wallis test with Dunn’s multiple comparisons test was used. For pairwise analyses, for example between 10 μM diflunisal and 10 μM compound 3b, the nonparametric Mann-Whitney *U* test was used. All statistical significances were expressed as two-tailed *p* values, where * means *p* = 0.01–0.05, ** means *p* < 0.01, *** means *p* < 0.001, and **** means *p* < 0.0001.

## Figures and Tables

**Table 1:** Univariate statistics for both HepG2 and Hep3B cells treated with compound 3b or diflunisal (control).

Metabolite	Retention Time (min)	Derivatives Formed	HMDB Number	HepG2	Hep3B
Ethanolamine	9.26	2TMS	00149	↓↓↓	
	12.94	3TMS			
Glycolic acid	9.97	2TMS	00115	↓↓	
Glycine	10.67	2TMS	00123	↑	
	13.54	3TMS			
Valine	12.18	2TMS	00883		↓
Leucine	12.99	2TMS	00687		↓
Isoleucine	13.32	2TMS	00172		↓
Proline	13.41	2TMS	00162		↓
Succinic acid	13.53	2TMS	00254		↓
Glyceric acid	13.83	3TMS	00139		↑↑
Uracil	13.96	2TMS	00300	↑↑↑↑	
Serine	14.24	3TMS	00187		↓
Threonine	14.62	3TMS	00167		↓
Aminomalonic acid	15.73	3TMS	01147	↓	
Erythritol	16.20	4TMS	02994		↓↓
Aspartic acid	16.32	3TMS	00191		↓↓↓
5-Oxoproline	16.40	2TMS	00267	↑↑↑↑	↓
Erythronic acid	16.67	4TMS	00613		↓↓
Cysteine	16.79	3TMS	00574	↑	↓
Creatinine	16.87	3TMS	00562	↓	↓
2-Oxoglutaric acid	16.96	2TMS; 1MOX	00208	↑	
Ornithine	17.44	3TMS	00214		↓
Glutamic acid	17.50	3TMS	00148	↑↑↑↑	↓
Phenylalanine	17.68	2TMS	00159		↓
*N*-Acetylaspartic acid	18.11	3TMS	00812		↓
Lysine	18.50	3TMS	00182	↑↑↑	↓
	20.82	4TMS			
Xylitol	18.78	5TMS	02917		↓
Glycerol 3-phosphate	19.19	4TMS	00126		↓
Glutamine	19.25	3TMS	00641		↓↓
Hypoxanthine	19.66	2TMS	00157	↑↑↑	
Galactose	20.61	5TMS; 1MOX	00143	↑	
	21.08	5TMS; 1MOX			
Tyrosine	21.02	3TMS	00158	↑↑	↓
Pantothenic acid	21.55	3TMS	00210	↑↑↑↑	
*Myo*-inositol	22.64	6TMS	00211		↓
Phosphoglycolic acid	22.72	3TMS	00816	↓	
Ribose	22.76	5TMS; 1MOX	00283	↑↑↑	
Oleic acid	23.46	1TMS	00207	↓	
Fructose 6-phosphate	24.59	6TMS; 1MOX	00124	↑↑↑↑	↑
Glucose 6-phosphate	24.71	6TMS; 1MOX	01401	↑↑↑↑	↑
Arachidonic acid	24.80	1TMS	01043	↓	
Sedoheptulose 7-phosphate	24.88	7TMS; 1MOX	01068	↑↑↑↑	
Uridine	25.63	3TMS	00296	↑	
Guanosine	28.38	5TMS	00133		↑↑

↑, ↑↑, ↑↑↑, and ↑↑↑↑ means an increase for compound 3b relative to diflunisal (*p* < 0.05; *p* < 0.01; *p* < 0.001; and *p* < 0.0001, respectively). ↓, ↓↓, ↓↓↓, and ↓↓↓↓ means a decrease for compound 3b relative to diflunisal (*p* < 0.05; *p* < 0.01; *p* < 0.001; and *p* < 0.0001, respectively). TMS means trimethylsilyl derivative. MOX means *O*-methyloxime derivatives of ketones and aldehydes. HMDB is the Human Metabolome Database [18].
